# Understanding cardiovascular disease in day-to-day living for African people: a qualitative metasynthesis

**DOI:** 10.1186/s12889-021-10781-1

**Published:** 2021-04-17

**Authors:** Seifu Nigussie Tulu, Nasser Al Salmi, Jacqueline Jones

**Affiliations:** College of Nursing, University of Colorado Anschutz Medical Campus, Aurora, CO USA

**Keywords:** African people, Cardiovascular disease, Culture, Health belief, Risk factor, Lived experience

## Abstract

**Background:**

Globally, cardiovascular disease (CVD) accounts for 45% of all chronic non-communicable disease deaths and 31% of all deaths. CVD has remained the primary cause of death in the world for the past fifteen years. Compared to other continents, CVD and its risk factors are highly prevalent in Africa, but the continent also displays a low-level of knowledge and awareness of CVD, and poor perception of its risk factors. Little research has been done on the connection between the daily lived experiences of African people and the high prevalence and poor perception of CVD and its risk factors on the African continent. The aim of this study is to provide an in-depth understanding of the daily, lived experiences of African people and the connections between these experiences and the prevention, control, and management of CVD and its risk factors.

**Methods:**

A systematic search was performed in PubMed, CINAHL, EMBASE, Psych INFO, and Web of Science databases to identify published English qualitative studies of CVD and its risk factors. Qualitative metasynthesis included structured techniques of data immersion and quality appraisal, thematic synthesis, and reciprocal translation.

**Results:**

Seven studies met the inclusion criteria. Four major themes were identified from the metasynthesis: 1) understanding and beliefs about CVD; 2) perceived causes/risk factors for CVD; 3) understanding and belief about obesity; 4) perceived treatment options for CVD. The metasynthesis identified a consistent disconnect among African people between seeing CVD as a deadly and chronic disease and their perceptions of the minimal signs and symptoms of the disease in the early stages. This was further compounded by the gap between traditional healers and health care professionals.

**Conclusions:**

Perceptions of CVD, its risk factors, and treatments were influenced by religious and cultural factors. Given the minimal signs and symptoms experienced in the early stages of the disease, there was a consistent disconnect among African people between seeing CVD as a deadly and chronic illness. Further investigations of the religious and cultural influences and educational programs related to these areas of disconnect are needed to improve the knowledge, attitudes, and beliefs of African people.

## Background

Cardiovascular disease (CVD) is a term used to classify many kinds of heart and blood vessel disorders, including congenital heart disease, coronary heart disease, cerebrovascular disease, peripheral arterial disease, rheumatic heart disease, deep vein thrombosis, and pulmonary embolism [1]. CVD accounts for 45% of all chronic, non-communicable disease (CNCD) deaths [2] and 31% of all deaths worldwide [1]. These deaths can be prevented by working on four major behavioral risk factors: unhealthy diet, tobacco use, physical inactivity, and the harmful use of alcohol that leads to the development of CVD.

The American College of Cardiology (ACC) and the American Heart Association (AHA) stated that the most important way to prevent CVD is to promote a healthy lifestyle such as healthy diet, physical activity, smoke exposure, smoking cessation, and moderate alcohol consumption [3]. A healthy lifestyle lowers CVD risk by 50% for high genetic risk persons and prolongs life expectancy [4, 5]. The practice and adherence of a healthy lifestyle are affected by various factors such as; individual’s beliefs, attitudes, knowledge, cultural issues, accessibility, availability, affordability, parental support, income, peer pressure, institutional/physical environment (neighborhoods, worksites, and schools), regulations, policies (local, state, and federal), health care provider counseling, absence of sign and symptoms, stress/anxiety, and social media [6–10].

Studies show a high prevalence of CVD and its risk factors in Africa compared to other continents [11–13]. The African Union report found that hypertension (HTN) is the second greatest health challenge on the continent after HIV/AIDS [14]. HTN prevalence is highest in Africa (46% of adults 25 years and above) and lowest (35%) in the Americas [15]. Obesity, a significant factor in the development of CVD, is high in the African continent [16]. There has also been reports of a high prevalence of physical inactivity in South Africa compared to other African countries and the global average [17]. Most studies based in Africa and on African immigrants living in non-African countries, showed a low level of knowledge and awareness of CVD and poor perception of its risk factors [18–21].

The high prevalence of CVD and its risk factors in Africa call for urgent intervention. Knowledge of the disease and how culture influences CVD is crucial for culturally sensitive management approaches. Individual beliefs, attitudes, practices, and cultural contexts affect unhealthy practices and behavior change. This qualitative metasynthesis provides a snapshot of the daily lived experiences of African people regarding CVD and its risk factors, a key to preventing, controlling, and managing CVD and its risk factors.

Health literacy is related to the use of health services and involvement in self-management of chronic conditions [22]. There is an association between low levels of health literacy and low educational level, low income, ethnic minority status, and living alone [23, 24]. These factors are very common in Africa. Low health literacy and risk perception are the possible causes of the increasing burden of CVD in Africa [20]. Therefore, the purpose of this metasynthesis is to explore African people’s perceptions of CVD and its associated factors in day-to-day living.

## Methods

### Overview

Qualitative metasynthesis of primary qualitative studies was conducted and is reported in line with established international standards [25–27].

The study used a process for qualitative metasynthesis [28, 29], which included a structured research question, an inclusive literature search strategy, a quality appraisal of the final selected articles as data immersion, and a metasynthesis of the findings using the techniques of thematic synthesis, reciprocal translation, and interpretive triangulation. The research question used to guide the search was, “How do African people understand CVD and the influences of cultural and religious beliefs on their view of CVD and risk factors in day-to-day living?”

### Search strategy

**Fig. 1:**
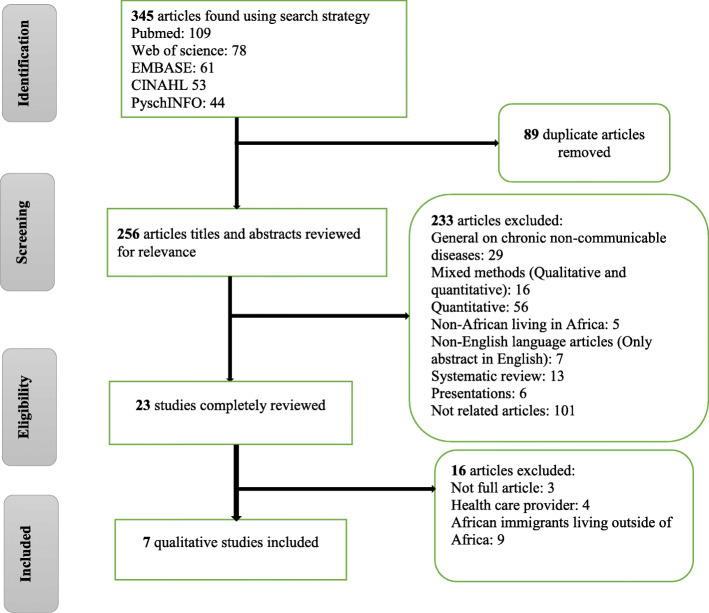
PRISMA flow diagram

The databases searched were PubMed, Web of Science, EMBASE, CINAHL, and PyschINFO during June–July 2018. In order to gather the historical perspective of cultural attitudes and perceptions, the search was not limited to a specified time. Only articles published in English were included for analysis. The search terms used were ‘cardiovascular disease,’ ‘unhealthy diet,’ ‘physical inactivity,’ ‘obesity,’ ‘smoking,’ ‘alcohol,’ ‘risk factor,’ ‘Africa,’ ‘health belief,’ and ‘qualitative.’ Original qualitative studies conducted in African countries with African people were included. Although CNCD shares four common risk factors (i.e. unhealthy diet, physical inactivity, smoking, and excessive consumption of alcohol), only studies that focused on factors associated with CVD were included. Articles that studied the underlying factors for these four common risk factors were also included. The studies on CNCD that presented CVD as one type of disease, studies on people from other continents living in Africa, studies on African people living in continents other than Africa, and studies on critically ill CVD patients were excluded. Studies on health professionals in Africa were not included since their medical knowledge could affect their cultural beliefs and perceptions. Non-published literature was not included. The search process following the Preferred Reporting Items for Systematic Reviews and Meta-analysis (PRISMA) guidelines is presented in Fig. 1.

### Data extraction and qualitative synthesis

A four-step qualitative metasynthesis was used [25]: 1) each article was taken as a whole as interpretive data; 2) data immersion and quality appraisal; 3) thematic synthesis [26]; and 4) reciprocal translation [28]. The information extracted after reading the articles was categorized under the following topics: study purpose, country of origin, study design, methods, participants, and main findings (Table 1).

**Table 1:** Summary of the seven included articles in the CVD and its risk factors qualitative metasynthesis

Study #	Author, Date, Country	Study purpose	Setting	Study Design	Methods	Participants
1	Ssewanyana et al., 2018, Kenya	To explore the perceptions of adolescents on unhealthy diet and sedentary lifestyle	Community of Kilifi county	Descriptive qualitative analysis	Snowball sampling; 10 FGD for adolescents, lasts for 75–120 min; In depth Interview for 10 adults, lasts for 60–90 min; transcribed verbatim; digitally recorded	*N* = 78 (68 adolescents (10–19 years) and 10 adults working with adolescents
2	Risenga et al., 2007, South Africa	To explore cultural values, beliefs and practices in relation to HTN	Community of Limpopo province	Exploratory, descriptive and contextual qualitative analysis	nonprobability purposive sampling; interview for traditional healers; FGD for patients with HTN; content analysis and Tesch’s stages of data analysis	*N* = 45 (30 patients with HTN and 15 traditional healers)
3	Temu et al., 2017, Kenya	To explore lay beliefs about HTN among HIV-infected adults	Kenyatta Referral and Teaching Hospital Comprehensive Care Center	Descriptive qualitative analysis	Purposive sampling; 6 FGD (5–8 participants), pretested in 10 in-depth-interview, PI and RA guide the FGD, last for approximate 90 min, audiotaped, groups added until inductive analysis revealed no new concepts, written notes and audio recording; Nvivo 11 software for analysis	*N* = 53 (31 F), age: ≥ 18 years, all had documented HIV diagnosis
4	Surka et al., 2015, South Africa	To explore the knowledge and perceptions of community members about risk for CVD	Community of Nyanga	Qualitative framework analysis	Purposive sampling; 3 FGD (8–10 participants), lasts for 60–90 min, digitally recorded; transcribed verbatim	*N* = 28 (24 F); age ≥ 25 years
5	Roos et al., 2015, South Africa	To determine the self-perception and behavior in relation to risk for IHD in a cohort of South African PLWHA	HIV clinic in Johannesburg	Qualitative descriptive/thematic analysis	Purposive sampling; interview, open-ended, tape recorded; descriptive analysis and conventional content analysis	*N* = 30 participants on HAART treatment for 6–12 months; age: 20–65 years
6	Okop et al., 2016, South Africa	To explore the perceptions of body size, obesity risk awareness, and the willingness to lose weight	Community of Langa	Descriptive qualitative analysis	Purposive sampling; 8 FGD (9–14 participants), lasts for 90 min, digitally recorded, notes taken; height and weight measured, and BMI calculated; Atlas.ti software for analysis	*N* = 78 (36 F), 34.6% obese, 24.4% overweight, and 41.0% optimal weight; age: 35–70 years
7	Namukwaya et al., 2017, Uganda	To explore the beliefs and understanding of their HF	Mulago National referral Hospital and patients homestead	Exploratory qualitative analysis	Purposive sampling; serial in depth interview (3 times); grounded theory approach; QSR Nvivo software	*N* = 21 patients with HF; age ≥ 18 years

*PLWHA* People Living With HIV/AIDS, *FGD* Focus Group Discussion, *F* Female, *N* Total number of participants, *HAART* Highly Active Antiretroviral Therapy

The quality attributes of the selected articles for the final analysis were assessed using a 17-item quality appraisal tool outlined by Letts and colleagues [29]. Each of the articles was evaluated with this tool. This evaluation provided an opportunity for the researchers to familiarize themselves with each article as a first step in data analysis. For data extraction, the whole article were used and read while for the sytnesis, immersion, reading, and rereading the whole article were performed. Each article was discussed by the team to determine if the study was relevant to the research question. The team had three members; one senior qualitative methodologist and PhD prepared professor, and two PhD students. Critical appraisal was used as a data immersion strategy as no consensus exists for the exclusion of qualitative studies from metasynthesis based on such appraisal, due to the wide variation in reporting requirements [30]. The quality appraisal of the articles can be found in the succeeding tables.

Using manual, team-based, inductive analytic techniques for thematic synthesis [26, 31] the authors initially reviewed each article individually. Guided by the research question, codes and beginning themes were identified for each article. The whole of the published article was considered the unit for data analysis as each study is taken as an interpretive act through publication. A line by line approach exploring similarities, differences, and novel insights was used. The team then met and discussed themes and associated sub-themes as a group. This iterative process of examining data within and across articles and engaging in interpretive triangulation across team members was repeated several times. Derived analytic themes and subthemes were compiled. The process of integrating and back-translating interpretations from one study to another is called reciprocal translation [28]. The final reciprocal translation summary of emerging themes can be found in Table 2. Finally, the team discussed how the findings lead to a deeper understanding of CVD in the daily lives of African people and how the findings might inform research and CVD education.

**Table 2:** Reciprocal Translation Table

Derived Analytic Theme/Subthemes	In paper # (as listed with Table 1)	Primary study themes (as labelled)
**1. Understanding of and beliefs about CVD**		
This theme is about how people interpret CVD and its risk factors		
Illness	2 (p.81), 3 (p.3), 4 (p.4), 5 (p.3), 7 (p.7)	Thick blood, high blood, anger of ancestors (#2); pressure, rushing of blood through the veins, blocking vein, fast flow of blood in heart, blood clot (#3); heart problem, stroke (#4); heart pumping fast, heart can’t relax (#5); heart tired, abnormal heart vessels, scarred heart vessels, blood doesn’t flow, cracks on heart, blood leaking on heart, heart surrounded by water, water in abdomen, symptom that prevent someone working, experiencing symptoms continuously for more than 3 months (#7)
Signs and symptoms	2 (p.81), 3 (p.3), 5 (p.4), 7(p.9)	Protruding blood vessels, collapse, faint, failure to see properly (#2); dizziness, headache, seeing black (#3); swelling, sweating, chest pain (#5); heavy heart, menstruation, pregnancy (#7)
Prognosis	2 (p.82), 3 (p.4), 7(p.9)	treated successfully, completely heal (#2); it can clear, fatal, never wake up (#3); cure, improve (#7)
**2. Perceived causes/risk factors of CVD**		
This theme is about the causes of CVD that were considered by the population		
Stress	3(p.3), 4(p.4), 5(p.3), 7(p.9)	Getting angry, thinking too much, worrying too much, disagreement, being alone (#3); talking too much, get lost in thought (#4); keeping problems to themselves, crazy, unemployment, buy medication, buy food (#5); lots of problems, fright (#7)
Unhealthy diet	1(p.4, 6), 4 (p.5), 5(p.4), 7(p.13)	Junk foods, bottle of juice made from chemical, traditional meals sugar dense confectionaries, fast foods, drug and substance use (#1); nutritious foods, poverty, portion size (#4); too much fat eating (#5); too much spices (#6); raw salt, packed drinks (#7)
Physical inactivity	1 (p.7), 6(p.10),	Domestic chores, social media, body image, drug and substance use, health complications, parental practices, peer influence, gambling, paternalistic culture, school attendance, motorized transportation, public policy, card games(#1); chasing the air, exercise, crime rate, lack of facility and place (#6)
Religion	2(p.81), 3(p.4), 4(p.5), 6 (p.5), 7(p.13)	communication with ancestors (#2); will power (#3); ‘dark spirits’, ‘deity’, betond control (#4); God creation (#6); evil charms, evil curses or spells, witch craft, lack of faith in God, God test of the heart, Satan (#7)
Smoking and drinking alcohol	1(p.4), 6(p.5)	Drug and substance use (#1); weight loss, weight gain (#6)
**3. Understanding of and beliefs about obesity**		
This theme is about how community talk about obesity and its cause.		
Language of obesity	3(p.4), 6 (p.5–8)	Big people (3#), big bone, large body, large size, too much fat, cloth size (#6);
Perceived cause of obesity	6 (p.5),	Too much oil, rice and potato, fried foods, creation of God, culture, stress, socioeconomic status, too much spices, cow liver, high acidic food (#6)
Cultural attitudes toward obesity	6(p.5, 6),	Respect, happy, attractive, lot of money, no problem, eat nicely, eat a lot, affluence, laziness, tiredness, drowsiness (#6)
**4. Perceived treatment options for CVD**		
This theme is about interventions perceived and practiced in the community for the management and treatment of CVD.		
Modern treatments	2(p.81), 7(p.12)	Western medicine (#2), conventional medicine (#7)
Traditional medicines/Traditional healers	2(p.81), 3(p.5), 7(p.12)	Remedies, herbal medicines, throwing of bones, relationship with ancestors, ceremonial sacrifice, scarification, dreams (#2); conventional medicine (#3); witch doctor, traditional birth attendant, alternative medications, give me energy, deal with strong medicnes (#7)

## Results

The initial search strategy identified 345 publications. After title and abstract screening, 256 articles were obtained (Fig. 1). Seven articles were included for final analysis. These studies included a cumulative total of 333 participants. More than half (57.1%, *n* = 4) of the studies were conducted in South Africa, with two studies in Kenya and one study in Uganda. Most of the studies collected data via focus group discussions. Two studies, one in Kenya [32] and one in South Africa [33], explored the ways in which HIV infected patients in Africa understood CVD and its risk factors. One other study in Kenya also included HIV infected participants as one group of participants [34]. Studies targeted HIV infected participants since HIV is the most serious health challenge in Africa. People living with HIV are at increased risk of CVD since immune response and HIV-related inflammation were found to be risk factors for CVD [35, 36]. Four studies [32, 33, 37, 38] were mainly focused on the CVD while the other three [20, 34, 39] primarily focused on the risk factors for CVD. A summary of the seven articles is presented in Table 1.

The metasynthesis identified a consistent disconnect among African people between seeing CVD as a deadly and chronic disease and their perceptions of the minimal signs and symptoms of the disease in the early stages. This was further compounded by the gap between traditional healers and health care professionals. Four themes emerged that describe the ways in which African people view CVD and its risk factors in day-to-day living. The themes included: 1) understanding and beliefs about CVD; 2) perceived causes/risk factors for CVD; 3) understanding and beliefs about obesity; 4) perceived treatment options for CVD (Table 2).

### Theme 1: understanding and beliefs about CVD

This theme describes the ways in which African people understand and perceive CVD. The theme also describes ways in which African people understand and view the biomedical model of care. The ways in which people perceive CVD and its main types, including; myocardial infarction (MI), cerebrovascular accidents (CVA), HTN, and chronic heart failure (CHF), were discussed in terms of culture, custom, religion, individual perspectives, and health care provider information misperceptions. Participants’ interpretation of CVD along with signs and symptoms and the association of the signs and symptoms related to CVD were also identified. Three subthemes were derived from this theme: illness, signs and symptoms, and prognosis.

#### Illness

The definition of illness that participants, families, and the community expressed in interviews [37] included: 1) having symptoms that prevent someone from working and participating in the activities of daily living; 2) experiencing symptoms continuously for 3 months; 3) having symptoms visible to others [32] such as swelling of body; 4) developing symptoms of disease that the community fear such as tuberculosis; 5) developing symptoms that were acute and severe such as breathlessness; 6) experiencing symptoms not responded to by local remedies.

Most participants did not know about CVD, and they claimed that it is the doctor’s responsibility to educate them about the disease. One participant from South Africa said,

“… We do not know these diseases because the doctor does not explain” [20]. Names for CVD in local languages may or may not have the same definition as medical terms, which affected participants’ understanding of the disease. In South Africa, Limpopo Province, HTN was defined as ‘having a high amount of blood’ and was named “Ngatileyikulu” and “Nómbe” in the Tsonga language [38].

Additionally, most participants gave a detailed explanation of MI as “heart attack” and CVA as a stroke, but they were unable to define and explain HTN [20, 37]. One participant mentioned, “They say I have high blood pressure and I am taking these tablets, which I do not know what they are for” [20].

Participants also stated there was little that could be done to prevent HTN [32]. Behavioral changes were not perceived as prevention strategies for CVD.

“Only the hospital can make the heart disease right … A person can’t help themselves; they can’t do something; the hospital must help” [33].

HTN was thought to be a disease that everybody experiences in life. The commonly-reported acute complications of HTN were sudden death, paralysis, blindness, and heart attack. The acute complications—including kidney disease, nosebleed, liver disease, emotional disturbances, headache, and pancreatic disease—were rarely mentioned [32].

In-depth interviews of 21 patients [37] found only one participant’s understanding of heart failure (HF) matched the medical notes recorded by health professionals. Participants expected stroke occurrence only among persons who were ill or had known HTN. Due to this belief, some participants were dubious when someone they considered healthy was diagnosed with a stroke [20]. These findings indicated the conflict of participants’ beliefs with those of the biomedical model. Differences in both patients and health care providers’ beliefs, likely hinder the effective treatment of HTN, including medication adherence [20, 37].

#### Signs and symptoms

The signs and symptoms of CVD have cultural and religious meanings, interpretations, and interventions [38]. The majority of participants did not know about the signs and symptoms of HF, and they associated the signs and symptoms they were experiencing with menstruation, pregnancy, aging, HIV, and with the side effects of HIV medication. A cough was regarded as a simple symptom that comes and goes. Finding appropriate words in local languages to fully describe the medical terminology and pathophysiology of CVD to patients resulted in misinterpretation of the diagnosis. Participants’ health literacy levels also affected the understanding. Terminologies, such as “cracks on my heart,” “blood was not flowing,” and “surrounded by water,” were commonly mentioned to describe their illness [37].

The commonly-mentioned signs and symptoms of HTN were continuous headaches, migraine in the forehead, dizziness, painful body, swelling, failure to see properly, seeing black color, protruding blood vessels, tiredness, nose bleeding, collapse, and feeling faint [32, 38]. Although participants did not mention the correct number of blood pressure readings, they were eager to learn the readings [32]. The common signs and symptoms of Ischaemic Heart Disease (IHD) mentioned by participants were chest pain, sweating, and breathing difficulties [33].

#### Prognosis

The majority of participants viewed CVD as a curable and preventable disease. Even though HTN was considered more serious and fatal than HIV, there was a common belief that HTN was an asymptomatic and temporary disease [32]. The view that HTN was a curable disease with limited symptoms led participants to ignore/refuse any medications for HTN that were taken for a long duration [32, 38]. Similarly, the majority of participants thought HF was a curable disease.

“As usual I expect them to cure the problem” [37].

There were three views on IHD. The majority of the participants described IHD as preventable, while others described it as unavoidable; others were unsure about the prognosis of IHD [33].

There was also a group of participants who understood the chronicity of CVD but expected improvement. Elder individuals believed that once they contracted an illness at their age, the illness would stay with them until death [37].

### Theme 2: perceived causes/risk factors for CVD

This theme is about perceived causes/risk factors for CVD. The five subthemes under this main theme include: 1) stress; 2) unhealthy diet; 3) physical inactivity; 4) religion; 5) smoking cigarettes and drinking alcohol.

#### Stress

Stress was the most frequently stated perceived cause of CVD in most of the studies [20, 32, 33]. Participants mentioned several causative factors (stressors) that cause stress. These included worrying or thinking too much [20, 37], worrying about their HIV diagnosis or about their family/children [33], emotional distress from the negative response of a child being disciplined [20], tension, anxiety, anger, being alone, unemployment, keeping problems to themselves [33], and disagreements [32]. Stress management was mentioned as the method of controlling and curing HTN [32]. Although stress was the most commonly mentioned risk factor for CVD, only family support was stated as a coping strategy to manage stress [33].

#### Unhealthy diet

Participants mentioned that what they ate resulted in CVD [32, 33]. Traditional foods were defined as foods that were made from locally grown cereals, fruits, and vegetables and which were consumed by ancestors [34]. Since traditional foods were made from fresh, garden ingredients, and since CVD was not common among ancestors who mainly consumed traditional foods, such foods were considered healthy foods by most of the participants [20]. Participants discussed the problem of eating monotonous traditional foods daily because of the expenses of purchasing a variety of food [34].

Adolescents strongly preferred non-traditional foods (i.e. processed and packaged foods and drinks) such as deep-fried potatoes, potato chips, commercially processed juices, cakes, sweets, and ice cream, due to the taste and method of preparation of non-traditional foods, and due to the association of traditional foods with low social status. A 30-year-old on the government staff of Kilifi County, South Africa explained:

“It gives them [adolescents] a feeling that if you are a modern person of this era you have to take things like ice-creams and the sugary foods not knowing that its almost opposite with your health” [34].

Factors that contributed to the consumption of unhealthy foods included access to cash, lack of parental and school monitoring, poor regulation of food products and hygiene products, urbanization, large family size, and the accessibility and the low cost of junk foods. Poor appetite caused by the use of drugs and substances such as khat (a psychostimulant plant believed to be originated in Ethiopia) [40] and cigarettes among adolescents also led to poor dietary behavior [34].

Foods with excessive salt and fat were the ones most commonly mentioned as the cause of CVD [32, 37, 38]. But, for making the taste of meat better, fat can be added to the meat [37]. Even though participants mentioned salt as a risk factor for CVD, they perceived only raw salt as a risk factor [32, 37]. Participants mentioned eating crystals of raw salt to be the cause of their HF [37]. Red meat and cooking oil were also stated to be a cause of CVD [32, 33].

Study participants misinterpreted the advice of health professionals. They tried to limit their fluid intake, believing/understanding/ that they were advised to do so and that limiting fluid was a good self-care method.

“—Now with the heart, they tell us to eat fruits, we should not eat salt, no water; this illness is so selective” [37].

“They tell us to drink less but remember the other medicine that they give us dries the throat you have to drink when you take it, so it is a bit difficult to balance the two, you have to be careful. Because at my age I cannot eat a lot I drink more” [37].

In rural settings, healthy foods such as vegetables, fruits, and cereals are more accessible, and, unless there are poor farming practices, are cheaper than unhealthy foods [34].

Poverty was mentioned as a factor influencing the consumption of unhealthy foods, including the consumption of whatever was available and affordable [20, 34]. In contrast to this, wealthy households were more likely to consume expensive, unhealthy foods such as cakes and ice cream [34]. Poverty was also stated as a major factor that made participants feel unable to change their unhealthy eating [20].

#### Physical inactivity

Few participants mentioned physical inactivity as a risk factor for CVD [32, 33]. Taking a physical education course in school and the existence of sports clubs allowed students to participate in physical activity [34]. However, many factors hindered participation in physical activity. These factors included: sitting long hours to gossip or hangout; gambling (including card games); using mobile phones (game and chat); watching television, videos, and movies; being underweight; taking medication; lack of interest in physical activity; experiencing academic pressure; having romantic relationships; experiencing peer influences; having access to motorized transportation; having health conditions (such as asthma); being affected by parental practices (such as not allowing children to play sports and punishing them when they did); and believing physical activity to be a waste of time that leads to dirtiness [34].

“So many people chat to an extent that they end up forgetting about exercising. That is because they are always busy with their phones chatting” [34].

Drug and substance use were also mentioned as it affected physical activity and led to dizziness, weakness, and sleepiness [34]. Working too much was also considered the cause of HF [37].

#### Religion

Religion was mentioned as a cause/risk factor for CVD. Participants’ religion was also seen to play an important role in the diagnosis, control, and management of CVD. The illness was perceived as a test of faith in God. It was also believed that Satan causes illness while God brings recovery [37].

People associated the signs and symptom of CVD, such as swelling, with evil curses or spells. Deity, witchcraft, evil charms, and “dark spirits” were mentioned as a cause of CVD by traditional religious believers, especially when the individual experienced swelling of the body [20, 37].

Participants mentioned the presence of continuous communication with their ancestors as a source of success in their life. Participants explained that causing the ancestors to get angry was a cause of illness. Such anger is brought about by a breach of taboos and confidentiality. Participants would report all events in the family to their ancestors [38].

Being obese or thin was considered to be an act of God [39]. Participants stated that they had no ability to control their CVD once diagnosed unless God controlled it.

“It is only God who can help us” [20].

Taking medications and seeking health care were considered to be evidence of a lack of faith in God [37].

#### Smoking and drinking alcohol

A study by Temu et al., 2017 found that 23% of participants were smokers and 23% were alcohol users. However, none of the participants mentioned tobacco use or alcohol consumption as a risk factor for HTN [32]. A study in South Africa found participants also failed to mention smoking as a risk factor for CVD [33]. There were also participants who were aware of the risk of tobacco smoking and excessive alcohol consumption. Interestingly, participants who did not drink alcohol became skeptical when they were diagnosed as having the disease [20].

Smoking and alcohol consumption led to poor appetite and poor motivation to exercise [34]. In contrast to traditional foods, traditional alcohol (alcohol made at home from locally available products) in Africa was perceived as being more harmful than modern alcohol. Ways of drinking alcohol adding a mixer, such as spirits, was perceived to reduce the harm.

“If I am drinking, I must drink like white people, because when they are drinking, they dilute their alcohol [spirits], unlike us; we just drink it raw as it is [without the addition of a mixer]” [20].

The other perceived causes/risk factors for CVD included: HIV, children’s noise [37], the anger of ancestors [38], depression [20], getting angry, getting shocked, hot pepper, antiviral drugs, arthritis medications, coffee, and Viagra [32].

“… Also these children who play and shout or make noise they frighten you and then you get palpitations” [37].

There were also participants who mentioned nonadherence to antiretroviral (ARV) drugs as a possible cause of HTN [32], and poor adherence to anti-hypertensive drugs as a risk for stroke [20]. Physical injury during childhood and perceptions of physical injury to the heart by babies in utero were also mentioned as a cause of CVD.

“… when the baby was turning in my womb he kicked my heart” [37].

Participants who experienced HTN in their family perceived that they inherited the disease from their family [32]. Some participants perceived heredity as the second causative factor for CVD next to stress.

“Like in my case …. I have not experienced something bad to cause me stress …. I think my mother passed it to me she had pressure” [32].

Age was mentioned by some participants as a risk factor for CVD [37], but was not mentioned as a risk factor by other participants [32].

### Theme 3: understanding and beliefs about obesity

This theme is about the ways in which African people perceived obesity and its causes, and the ways in which this informs our understanding of CVD risk factors. The three sub-themes under this theme are: 1) language of obesity; 2) perceived causes of obesity; 3) cultural attitudes toward obesity.

#### Language of obesity

Most participants used the word “fat” to refer to obesity. When participants needed to differentiate obesity from being overweight, they used the phrase “too much fat.” They also used “normal fatness” to refer to normal weight. For example, a man who had optimal weight described obesity in the following way:

“Too much fat is caused by what we eat-like junk food” [39].

Other phrases associated with obesity include “big people” [32, 39], “big bones,” “large body,” and “large size” [39].

#### Perceived causes of obesity

The commonly mentioned causes of obesity were physical inactivity, hereditary, lifestyle, and an unhealthy diet (such as the consumption of excess fat, red meat, oil and starch, and fried or junk foods.) “Overweight or skinny—we were created by God to be the way we are” [39]. Stress, socioeconomic status, age, poor access to fruits and vegetables, the consumption of too many spices, the consumption of cow liver, and the consumption of highly acidic foods were also mentioned as a cause of being overweight/obese [39].

#### Cultural attitudes toward obesity

Culturally, African women were expected to be overweight. Most female participants also expressed a strong preference for a larger body size. Being overweight/obese was believed to be a sign of respect/dignity, good health, happiness, and wealth. However, obesity at an older age was not considered healthy. The majority of overweight/obese participants perceived their weight as normal or medium [39]. This belief may have caused some participants to fail to mention obesity as a risk factor for CVD [20, 33]. Being overweight was also not considered a risk factor for CVD, and women who were overweight were not willing to lose weight. Even participants who experienced illness because of their previous weight, still desired to gain weight. One woman who was overweight said:

“I would like to gain more weight … As I have mentioned before, I was weighing 63 kg before I got sick, today I can see that I weigh 49 kg. This weight is not making me happy at all. I would like my weight to be at least 60 kg” [39].

There was also a group that associated fatness with laziness, tiredness, and drowsiness [39]. Participants noticed that the majority of CVD patients they encountered were “big people.” They started to consider CVD as the disease of “big people” [32, 39]. Men were more concerned about the risk of obesity than women [39].

There was a universal opinion that viewed thin individuals as unhealthy, as sufferers of diseases such as cancer, tuberculosis, and HIV/AIDS, and as sufferers of stress and unhealthy eating. Due to such views, thin people were stigmatized. “If you are skinny you are not healthy. When you are thin, people think you have HIV or TB” [39]. There was also a contradicting opinion which viewed thin people as attractive and smart.

The strategies to lose weight mentioned by participants were smoking, slimming medications, reduced consumption of fatty and starchy food, and physical activity. Although the problem of accessibility of healthy food was mentioned, the consumption of healthy foods such as fruits and vegetables were also cited as another method of weight management [39]. The barriers to physical activity mentioned were a lack of facilities and places for exercise, domestic chores for women, poor perceptions and motivation for exercise among women, and cultural resistance to jogging in the street and associated crime. Female body image perceptions such as reticence to expose body parts during physical activity and the feeling of embarrassment for being overweight, were also perceived barriers to exercise. An overweight woman expressed women jogging in the street as:

“… some women run in the street as if someone is chasing them. I don’t like to chase the air, though chasing that air, I’m told can help make you fit” [39].

Weight loss was assessed by clothing size, since self-weighing at home and clinic visits for weight measurement was not common [39].

### Theme 4: perceived treatment options for CVD

This theme is about interventions perceived and practiced in the community for the management and treatment of CVD. The two subthemes of this theme are modern treatments for CVD and traditional medicines/traditional healers.

#### Modern treatments for CVD

Modern treatments refer in this context to therapeutic procedures and drugs ordered for CVD by healthcare professionals. Modern treatments were used when traditional medicines did not improve a patient’s condition [37]. Participants perceived the medications for CVD as “western medicine.” Western medicine was used for complications which were beyond the ability of a traditional healer, such as stroke [38]. Western medicines were not preferred since these medications were believed to be taken for a long duration [38]. Since participants viewed HTN as a curable disease, they preferred medications and practices that gave them hope to be cured [32, 38].

There were barriers identified that affected the use of modern treatments. Participants did not want to take medicines on an empty stomach, and reported skipping doses if they were unable to buy food [33]. Some participants who were on ARV drugs considered themselves healthy and not at risk for CVD, since they were taking medications [33]. Factors that made participants not adhere to medications were perceptions that injectable medications were more effective than oral medications, and that taking medication for a long duration made the medications not work as effectively, or made the body develop a tolerance to them [37]. There were participants who took willpower as the management strategy for CVD and refused to take medication. Other reasons mentioned for refusing to take CVD medications were; taking too many medications, especially if taken together with HIV medications, drug side effects, cost, availability, and a lack of knowledge about CVD [32]. Participants also used herbs and traditional treatments to decrease side-effects of their CVD medication and to enhance the strength of injectable medicines [37]. Taking too many pills was perceived to be the cause of cancer [20]. Participants who were on HTN and HIV medications preferred not to take antihypertensives when they felt the “pressure.”

“…. I refused medication because there is no way I can take the medication for the virus and then add for pressure … that is too much drugs in your body. I told the doctor …. I will control my pressure because there is a way you can feel it is high” [32].

“… my sister and mom take medications daily …. I have refused to take drugs completely … so for me I believe it can clear, it all depends with how you handle issues” [32].

Medications that reduced symptoms were perceived to be the most useful, and participants preferred to take them selectively. Due to a lack of health literacy, such selectivity resulted in choosing the wrong medication when participants were on multiple medications. Patients explained these as following:

“If I have those water tablets, you can deny me any other tablets but give me those tablets because they give a lot of relief” [37].

“These are the ones which help (points to ciprofloxacin) they help me pass a lot of urine, those (furosemide) were given to me but did not do anything” [37].

Misunderstanding their medical treatment also made some participants anxious. Participants believed that paracentesis would cause more fluid to accumulate in their stomach. Since participants associated oxygen therapy with people in the process of dying, these therapies worried them [37].

Some facilitators for using modern treatments for CVD were discussed. Participants’ families mentioned that they realized the signs and symptoms of HTN, such as a headache and dizziness, occurred if the patient did not take the proper medications [32]. The non-medical strategies used for the management of HTN mentioned by participants were sleep, exercise, water, milk, banana, and a diet with less fat and salt [32, 33].

#### Traditional medicines/traditional healers

Traditional medicines were used when modern treatments did not improve the condition of the participants [37]. Traditional healer management of the disease was similar to medical symptomatic management, since traditional healers treated the disease based on signs and symptoms. Liquid herbal medicines were used for tiredness and excessive sweating, dry roots of herbal medicine were used for a headache, burned molds and herbal medicine were used to loosen blood, which is believed to be high and thick, steam inhalation was used for cleaning blood, and ground herbal medicines mixed with fat were used to rub affected parts after a stroke [38]. These remedies were not used for all people. Steam inhalation was not used by people with stroke for a fear of causing collapse [38]. Tea and garlic were mentioned as the herbal remedies for CVD [32]. Some herbs were given a name that coincided with their purpose, such as “heart of the soil,” which was used to treat the heart [37].

Traditional healers stated that they scarified and applied herbal medicine to better manage HTN. Fear, pain, and agony were mentioned in relation to these practices. Some participants witnessed complete healing after traditional healer interventions [38]. However, participants recommended that traditional healers and healthcare professionals work together [38]. Bone-throwing and dream interpretation played an important role for the participants in diagnosing and finding treatments for HTN. In the case of bone-throwing, the diagnosis was based on the position of the thrown bones. A bone can be thrown to ask ancestors to identify the herbal medicine that works best for a patient. In dream interpretation, ancestors provide the diagnosis and treatment [38].

Traditional treatments and herbs were also used to remove evil curses or spells. Traditional religious believers sought care from witch doctors [37]. Since bad communication with the ancestors was considered a cause of illness, ceremonial sacrifice (with relatives) by slaughtering animals was utilized for the treatment of CVD [38].

When pregnant women experienced signs and symptoms of CVD, they sought help from a traditional birth attendant (TBA). TBAs associated the signs and symptoms of CVD with the size of the baby or amount of amniotic fluid TBAs often gave reassurances to pregnant women not to seek medical care.

“During the eighth month of pregnancy, I felt so heavy. I could not even lift a pot of food and put it on the fire, everything I did my heart would beat so much. There were some women, the traditional birth attendants I went to one of them and explained my situation and she said, no it is okay the child has a normal lie in the abdomen but might be big or is seated in a lot of water. So, I thought it was the pregnancy” [37].

## Discussion

This metasynthesis of qualitative studies presented the ways in which African people perceived CVD and its risk factors, which resulted in a beginning understanding of the cultural and religious beliefs of African people on CVD and its risk factors. Understanding the lived experiences and local cultural and religious beliefs of African people can help health care providers to more effectively prevent, manage, and control CVD and risk factors for CVD. There were contradicting perceptions about CVD and its risk factors, although the majority of these perceptions were not what the biomedical care model recommends.

The definitions of illness, such as having symptoms continuously for 3 months and having symptoms visible to others such as swelling of the body [37], contradicted the medical definitions and made participants seek health care after complications occurred and the condition became worse. HTN, which is called a “silent killer” [15], is perceived as a symptomatic, temporary and curable disease, especially if a person handles the symptoms effectively [32]. A study done in Tanzania supports this finding since patients with HTN that attended the emergency department held a belief that HTN is a curable disease [41]. This belief results in poor medication compliance and increases the risk of HTN-related complications, and there needs to be further investigation and intervention, such as educating the community.

Among African people, the most commonly reported perceived cause of CVD was stress, and there are studies that support this belief. Psychological stress is known to result in CVD morbidity and mortality [41–46], and traditional stress reduction strategies can reduce CVD recurrences and death [47–49]. There is a CVD type named stress cardiomyopathy (also called takotsubo cardiomyopathy or broken heart syndrome) because stress is the cause of the disease [50]. Therefore, exploring the knowledge and practices of African people regarding stress, need to be encouraged.

A majority of the participants mentioned that they experienced extreme stress for a long time and faced the problem of accepting the status of their HIV [32]. This may help health care providers understand how to handle positive results. Since new HIV infection prevalence is highest in Africa, extra care needs to be taken when health care professionals inform patients of the results of these screenings.

Adequate fluid intake is necessary for the body to be healthy [51–53]. This metasynthesis revealed that participants were often limiting their fluid intake [37]. Participants misunderstood the information provided by healthcare professionals about fluid intake and CVD symptoms.

Physical inactivity was also not recognized as a risk factor for CVD. There were also numerous factors that affected the physical activity of women [39] and adolescents [34]. The reasons family members chose for preventing children from participating in sports needs to be further investigated. Attitudes and misconceptions toward physical activity may be a factor, since physical activity was perceived as a waste of time.

Cultural support for African women to have a large body size coupled with an interest in being overweight and obese contributed to the high prevalence of these conditions. As a result of this, African women are at a higher risk for CVD. Other studies done in African countries found that cultural beliefs and opinions surrounding obesity included viewing obesity as a sign of respect, dignity, confidence, happiness, and wealth, and conversely, included the view that thinness was a sign of ill health [54–57]. Similar to the findings of this metanalysis, Spanish Roma population had alternative self-perceptions of their body size [58]. Different studies in African countries such as Nigeria [59], Cameroon [60, 61], and South Africa [62] found a higher prevalence of obesity in women than in men. Food insecurity also played a crucial role in the prevalence of obesity in Africa, even though affluence and excess consumption seemed to be a cause [63, 64]. Multiple factors contradicted the recommendations of the biomedical model, including cultural attitudes that viewed obesity as prestigious, women’s interest in being obese, and the practice of smoking to reduce their weight [39].

We found that there was a lack of acceptance and stigma toward thin people. A study from South Africa found that thin participants preferred to be overweight and therefore at risk for CVD, rather than be stigmatized by the community and considered to be an individual who had HIV/AIDS [65]. In contrast, in the US, overweight/obese people are stigmatized and discriminated against in the workplace, in school, and in healthcare settings [66–69].

Traditional medicine which has a long history, is still the major source of health care in the African continent where more than half of the population seeks treatment from traditional healers [70–74]. The World Health Organization (WHO) recommends facilitation of integration of traditional medicine into health systems and updated the *WHO Traditional medicine strategy 2002–2005* to 2014–2023 [70]. The close collaboration between traditional healers and health care professionals, along with an open discussion of both traditional and western medicines, may help in the effective management of CVD and its risk factors. The willingness of traditional healers to work with healthcare professionals will facilitate such collaboration. However, this collaboration may be hindered by the cultural beliefs, attitudes, and practices of patients, and also the attitudes and stereotypes of health care professionals toward herbal medicine and traditional healers.

The TBA’s understanding of CVD and its signs and symptoms is vital to prevent complications of the disease in pregnant women. TBA related rapid heartbeat with the big fetus and amount of amniotic fluid and reassured pregnant women not to seek medical help. TBA demonstrated knowledge gaps when diagnosing CVD based on the signs and symptoms [37].

Participants mentioned lacking food and skipping doses of medication [33], being unable to afford the cost of the medication [32], eating whatever was available and affordable [20, 34], and not eating healthy foods such as fruits and vegetables due to a lack of money [38]. But, participants slaughtered animals for ceremonial sacrifice [38]. These sacrifices affected the financial health of patients, preventing them from buying food and medicine, thus delaying the treatment of CVD and leading to CVD complications. For this reason, these ceremonial sacrifices need further investigation and exploration of alternative, acceptable practices.

The Individual and Family Self-Management Theory described by Ryan and Sawin [75] could be used to explain individual, family, social, and cultural considerations and interventions that result in short (proximal) and long (distal) outcomes. Self-management involves the use of knowledge and beliefs, self-regulation skills and abilities, and social facilitation for the management of chronic conditions. This study clearly identified the individual, family, social, and cultural considerations and interventions for the management of CVD and its risk factors. Some of the findings that support this include: 1) being alone, mentioned as a source of stress, and family support, one strategy perceived to manage stress; 2) cultural attitudes toward obesity that influenced women to be obese; 3) the social meaning of illness that causes participants to not seek timely health care; 4) social attitudes toward certain foods that made participants choose unhealthy foods. Therefore, this theory is the best fit to study health behaviors in African people in the future.

Misperceptions related to CVD and its risk factors were common on the African continent. A majority of African people in the studies perceived CVD and its associated risk factors, contrary to the biomedical model of disease management. There were several factors that promoted unhealthy behavioral practices on the African continent. These factors need the focus of health professionals working with African people regarding CVD and its associated factors. Knowledge of the beliefs, attitudes, and practices of African people is in many ways potentially more important than knowledge of the biomedical model of disease management. On the African continent, cultural contexts played a more important role than the biomedical model for the management of CVD and its risk factors. These disconnected beliefs, attitudes, and practices from the biomedical model affected health-seeking behaviors, changing unhealthy behaviors, and treatment compliance.

The metasynthesis focused on an interpretation of themes. As a first level analysis, a conceptual framework was not developed for this study. A broader data sample would enable a greater confidence in relationships. There are potential limitations to this metasynthesis. Since we did not access the original data, we may repeat the biases that other researchers created when doing their own interpretation. However, interpretations of interpretations are one aim of qualitative metasynthesis [76]. Studies of Africans in other languages such as French studies of Africans in Benin may have been missed. Since only studies done in three countries in Africa were found and used for this metasynthesis, this analysis may significantly underrepresent other African or regional understanding of cardiovascular disease in day-to-day living. There might be multiple other reasons underpinning the view of CVD and its risk factors and management beyond culture and religion among African people considering the limited number of studies. Unpublished data, theses and dissertations that can provide a rich data were not included.

## Conclusion

Perceptions of CVD, its risk factors, and treatments were influenced by religious and cultural factors. Knowledge of culture-related beliefs and practices is crucial for the effective management and control of CVD and its risk factors. There was a consistent disconnect among African people between seeing CVD as a deadly and chronic disease and their perceptions of the minimal signs and symptoms of the disease in the early stages. Further investigations of religious and cultural influences and educational programs on the areas of disconnect are needed to improve the knowledge, attitudes, and beliefs of African people. Most studies and participants highlighted stress as a common risk factor for CVD. Stress management strategies mentioned are important to alleviate the risk factor for CVD.

## Data Availability

Not applicable.

## Abbreviations

AHA: American Heart Association
ACC: American College of Cardiology
ARV: Antiretroviral
CVD: Cardiovascular Disease
CNCD: Chronic Non-Communicable Disease
HTN: Hypertension
US: United States
MI: Myocardial Infarction
CVA: Cerebrovascular Accidents
CHF: Chronic Heart Failure
HF: Heart Failure
IHD: Ischaemic Heart Disease
Kg: Kilogram
TBA: Traditional Birth Attendant
